# Varicella zoster-associated progressive lower cranial and upper cervical polyneuropathy: a case report

**DOI:** 10.1186/s13256-022-03484-7

**Published:** 2022-08-17

**Authors:** Fangzhi Jia, Eugene R. Ting, Joo Hyen Ahn, Andrew Duggins

**Affiliations:** 1 Concord Repatriation General Hospital, Concord, Sydney, NSW 2139 Australia; 2 University of Sydney, Sydney, Australia; 3 Department of Neurology, Westmead Hospital, Sydney, Australia

**Keywords:** *Zoster sine herpete*, Collet–Sicard syndrome, Polyneuritis cranialis, Cervical neuropathy, Case report

## Abstract

**Background:**

Multiple cranial neuropathies carry a wide range of differential diagnoses, and when combined with cerebrospinal fluid monocytosis they often suggest an infective etiology. Reactivation of varicella zoster virus has been associated with a wide range of neurological complications. Among the cranial nerves, the upper cranial nerves (trigeminal and facial nerves) are more commonly affected; there have been some reports of lower cranial polyneuropathies resulting from varicella zoster virus reactivation. However, polyneuropathy involving both the cranial and cervical nerves is rarely reported.

**Case presentation:**

This report highlights the case of a 64-year-old Chinese man presenting with an acute, severe dysphagia and dysphonia secondary to herpes zoster-associated progressive polyneuropathy involving the lower cranial and upper cervical nerves, without any mucocutaneous manifestations.

**Conclusions:**

To our knowledge, this is the first case of varicella zoster virus-associated cranial and cervical polyneuropathy in the literature. The report also highlights the poor sensitivity of varicella zoster virus DNA detection by polymerase chain reaction in the cerebrospinal fluid, and proposes that serology be routinely performed in such polymerase chain reaction-negative cases without a clear diagnosis.

**Supplementary Information:**

The online version contains supplementary material available at https://doi.org/10.1186/s13256-022-03484-7.

## Background

Infection of varicella zoster virus (VZV) has been associated with a wide range of neurological complications. It is a cause for polyneuritis cranialis, and tends to predominantly affect the upper cranial nerves (trigeminal, facial, and vestibulocochlear nerves). Lower cranial polyneuropathies, with or without rash, secondary to VZV infection or reactivation have occasionally been reported, and these cases highlight the tendency of VZV to involve multiple contiguous nerves [1–4]. Despite this, instances of VZV being implicated in polyneuropathies of both cranial and cervical nerves are rarely reported in the literature, if at all. This report highlights the intricacies in the diagnosis of a VZV-associated polyneuropathy involving the lower cranial and upper cervical nerves without rash, and discusses the mechanism as well as the advantages and disadvantages of the relevant VZV investigations in this scenario.

## Case presentation

A 64-year-old man of Chinese descent presented with a 3-day history of progressive and severe dysphagia, to both solids and liquids, associated with mild dysphonia and right-sided posterior shoulder pain. He had no odynophagia, dyspnea, fever, rash, constitutional symptoms, or other neurological symptoms. His medical history included only an episode of pneumonia 5 years earlier, and he took no regular medications. He worked as a butcher, was a smoker with a history of 50 pack-years, and did not consume alcohol. There were no significant occupational or environmental exposures. He was born in China and migrated to Australia in his mid-30s. There was no family history of neurological diseases. He had received the scheduled age-appropriate vaccinations in China, not including the chickenpox or varicella vaccines.

On examination on his presentation, he was afebrile and the rest of his observations were within normal limits (blood pressure 160/80 mmHg, heart rate 80 beats per minute, oxygen saturation 100% on room air). His speech was normal, and voice was not hoarse. Cranial nerves were intact, there was preserved motor and sensory function in all limbs, and he was normoreflexic throughout. Cardiorespiratory examination was unremarkable. His throat was not inflamed, with no obvious masses, goiter, or neck lymphadenopathy. He was able to swallow a small amount of water on sip testing, but appeared to have to force-swallow, and choked when trying to sip a larger amount.

A neurological examination 3 days later showed an absent right gag reflex, leftward uvula deviation, and absence of elevation of right side of palate with phonation (Additional file 1: Video S1). Laboratory results revealed a lymphocyte count of 0.8 × 10^9^/L, and the rest of his complete blood count, inflammatory markers, electrolytes, and liver and renal functions were normal. Urinalysis was unremarkable, and human immunodeficiency virus (HIV) and syphilis serologies were normal.

**Fig. 1:**
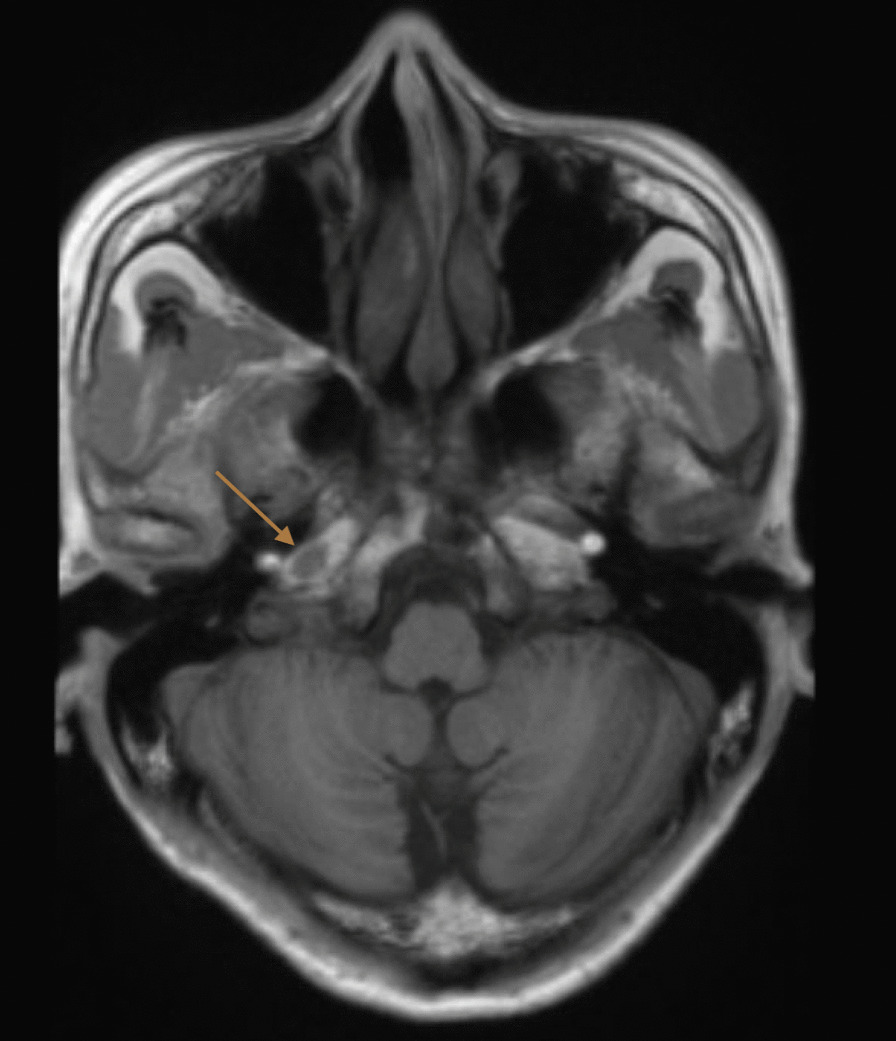
Magnetic resonance imaging showing an oval-shaped, nonspecific isointense T1 signal (arrow) near the right petrous apex

**Fig. 2:**
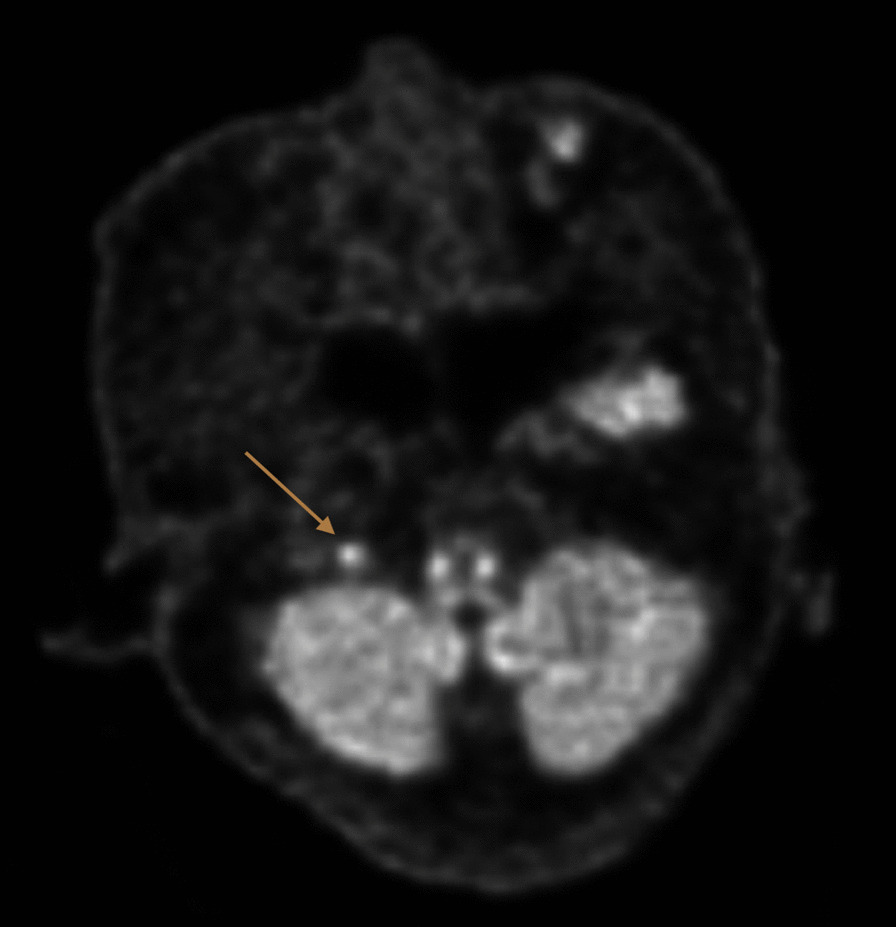
Positron emission tomography images showing increased glucose metabolism (arrow) in the right jugular foramen.

Computed tomography (CT) of the brain and cerebral angiogram were normal, and magnetic resonance imaging (MRI) of the brain showed a nonspecific oval isointense T1 signal near the right petrous apex measuring 4 × 7 mm (Fig. 1), with no associated T2 signal change or gadolinium enhancement identified. Whole-body positron emission tomography (PET) reported increased fluorodeoxyglucose uptake from brainstem into the right jugular foramen (Fig. 2).

His gastroscopy was unremarkable. Flexible nasal endoscopy revealed mucus pooling at the right vallecula with right vocal cord paresis. CT chest imaging showed bilateral emphysema and no evidence of lymphadenopathy or malignancy.

**Fig. 3:**
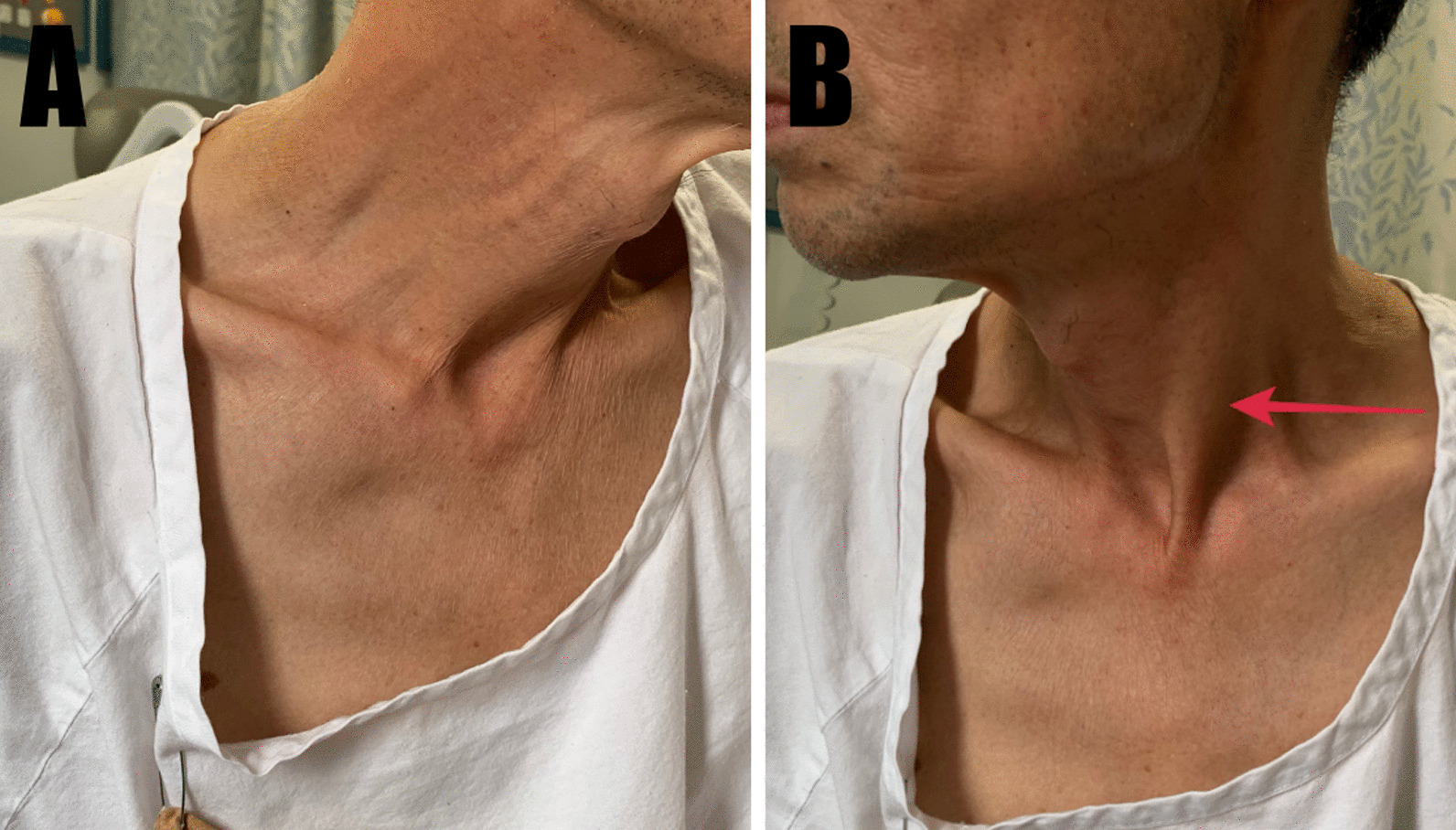
Atrophic right sternocleidomastoid muscle (**A**) compared to the left (**B**, arrow)

Over a period of 3 weeks, his dysphonia worsened, and he developed several new signs and symptoms: atrophy of right sternocleidomastoid muscle (day 10 since symptom onset, Fig. 3), leftward tongue deviation (day 16), and right-sided scalp dysesthesia extending from the right occiput to vertex associated with headaches (day 18). Cerebrospinal fluid (CSF) examination on day 15 after symptom onset showed a cell count of 63/mm^3^ (mononuclear:polynuclear cells, 60:2) with a negative viral and tuberculosis nucleic acid panel. Serum serologies for VZV were performed; the patient was positive for both VZV immunoglobulin M (IgM) and immunoglobulin G (IgG) on day 20, thus supporting a diagnosis of acute VZV-associated contiguous polyneuropathy.

He was commenced on intravenous acyclovir (10 mg/kg every 8 hours) for 7 days. His headache quickly resolved following initiation of antiviral treatment, and his dysphonia improved significantly. Minimal improvement in dysphagia was seen in the 2 weeks after antiviral treatment, and any oral intake was still not safe. He received a vocal cord medialization procedure on day 35, aiming to improve swallowing and voice and reduce aspiration risk; this provided only limited short-term benefit. He then underwent a percutaneous endoscopic gastrostomy tube insertion for long-term enteral feeding. At 6-month follow-up, his dysphagia had significantly improved with tolerance of thin fluid and soft dysphagia diet, and his VZV IgM titer was negative while IgG remained positive.

## Discussion and conclusions

Here we describe a case of migratory unilateral lower cranial and upper cervical polyneuropathy with lesion localization to the jugular foramen, with serology supporting the acute involvement of the varicella zoster virus despite the clinical manifestation of rash. To out knowledge, this is the first report of this kind in the literature.

The discernible progressive nature of the patient’s symptoms is noteworthy. Right-sided posterior shoulder pain, severe dysphagia, and mild dysphonia were the initial symptoms, followed by worsened dysphonia, new accessory neuropathy, subsequently hypoglossal neuropathy, and, lastly, occipital/parietal scalp dysesthesia. The scalp symptoms implicated the greater occipital and less occipital nerves, both of which originate from the C2 plexus. The clinical course was thus that of a progressive lower cranial (IX, X, XI, XII) and upper cervical polyneuropathy, sequentially involving the glossopharyngeal and vagus nerves (day 1 of symptoms), accessory nerve (day 10), hypoglossal nerve (day 16), and, eventually, C2 plexus (day 18). There was strong unilaterality throughout the course of his illness.

The combination of the clinical finding of progressive lower polyneuritis cranialis and C2 plexopathy, radiological lesion localization to the jugular foramen (which cranial nerves IX, X, and XI traverse), and the biochemical finding of serum lymphocytopenia and CSF mononuclear pleocytosis, suggests that this is a contiguous and progressive polyneuropathy initially localizable to the jugular foramen, likely of viral etiology. Unilateral palsies of the cranial nerves IX, X, XI, and XII is termed Collet–Sicard syndrome, which is one of the several eponymous lower polyneuritis cranialis syndromes.

Varicella zoster virus reactivation is a common cause of lower polyneuritis cranialis and contiguous polyneuropathies. Most cases are subtle and difficult to diagnose, compounded by the fact that many patients present without the characteristic rash for herpes zoster (*zoster sine herpete*) [2, 3]. The patient in this case had no cutaneous rash, and no vesicles were noted on otoscopic and flexible nasal endoscopic examinations. The mechanism for VZV contiguous polyneuropathy is thought to be transaxonal spread of the virus through interconnections between several ganglia [8].

Polymerase chain reaction (PCR) assays for VZV DNA performed on CSF have poor sensitivity in the setting of lower polyneuritis cranialis and other polyneuropathies associated with VZV reactivation. PCR positivity commonly occurs in the early stage of the disease (1–2 weeks) and is often not observed in episodes of reactivation [4, 9, 10]. VZV serologies in the serum and CSF are more sensitive compared with CSF PCR in the diagnosis of acute VZV infection and remain positive for a much longer period of time after the onset of symptoms [4].

Treatment of VZV-associated lower polyneuritis cranialis involves a course of antiviral therapy, often with adjunctive corticosteroids [1–3]. The clinical course is variable, with some showing excellent response while others unfortunately have long-term sequelae [2, 6]. Early treatment is key and should be initiated as soon as the diagnosis is considered [2], although diagnosis may be difficult and treatment is consequently often delayed.

In conclusion, our present case highlights the first case of VZV-associated lower cranial and upper cervical polyneuropathy in the literature. It shows that *zoster sine herpete* is an important cause of cranial and cervical polyneuropathy, the diagnosis of which is often missed as the sensitivity of PCR detection for VZV DNA in the CSF is low beyond the first week of disease.

## Supplementary Information

**Additional file 1: Video S1.** Lack of elevation of right side of palate on phonation.

## Data Availability

Not applicable.

## References

[1] Osaki Y, Matsubayashi K, Okumiya K, Wada T, Doi Y. Polyneuritis cranialis due to varicella-zoster virus in the absence of rash. Neurology. 1995;45(12):2293. 10.1212/wnl.45.12.2293. 8848213

[2] Mantero V, Rigamonti A, Valentini S, *et al*. Isolated acute dysphagia due to varicella-zoster virus. J Clin Virol. 2014;59(4):268–9. 10.1016/j.jcv.2014.01.008. 24529416

[3] Murata K, Miwa H, Kondo T. Polyneuritis cranialis caused by varicella zoster virus in the absence of rash. Neurology. 2010;74(1):85–6. 10.1212/WNL.0b013e3181c7da35. 20038777

[4] Skripuletz T, Pars K, Schulte A, *et al*. Varicella zoster virus infections in neurological patients: a clinical study. BMC Infect Dis. 2018. 10.1186/s12879-018-3137-2. PMC5970536 29801466

[5] Nomi N, Kodama S, Kawano T, Yoshida K, Watanabe T, Suzuki M. Varicella zoster virus infection involving lower cranial nerves: a report of three cases. Pract Oto-Rhino-Laryn. 2008;101(8):577–85. 10.5631/jibirin.101.577[InJapanese].

[6] Shi M, Zhou HW, Wang J, Zhang Y, Deng H. Analysis of two cases of varicella zoster virus antibody-positive jugular foramen syndrome. J Apopl Nerv Dis. 2018;35(3):268–70 (**in Chinese**).

[7] Tecellioglu M, Kamisli S, Erbay MF, Kamisli O, Ozcan C. A rare presentation of cranial polyneuropathy without rash caused by varicella zoster virus. Med Arch. 2017;71(4):293–5. 10.5455/medarh.2017.71.293-295. PMC5585840 28974853

[8] Letchuman V, Donohoe CD. Brainstem and cerebellar involvement in Ramsay Hunt syndrome. Case Rep Otolaryngol. 2019. 10.1155/2019/7605056. PMC6931020 31915561

[9] Ono N, Sakabe A, Nakajima M. Herpes zoster oticus-associated jugular foramen syndrome. Brain Nerve. 2010;62(1):81–4 (**in Japanese**). 20112795

[10] Nagel MA, Cohrs RJ, Mahalingam R, *et al*. The varicella zoster virus vasculopathies: clinical, CSF, imaging, and virologic features. Neurology. 2008;70:853–60. 10.1212/01.wnl.0000304747.38502.e8. PMC2938740 18332343

